# Endoscopic management of upper tract transitional cell carcinoma

**DOI:** 10.4103/0970-1591.65382

**Published:** 2010

**Authors:** James A. Forster, Victor Palit, Anthony J. Browning, Chandra Shekhar Biyani

**Affiliations:** Department of Urology, Castle Hill Hospital, Hull and East Yorkshire NHS Trust, Cottingham, East Yorkshire HU16 5JQ, UK; 1 Department of Urology, Pinderfields General Hospital, Mid Yorkshire Hospitals NHS Trust, Aberford Road, Wakefield, West Yorkshire WF1 4DG, UK

**Keywords:** Endoscopy, minimally invasive therapy, transitional cell carcinoma

## Abstract

Upper urinary tract transitional cell carcinoma (TCC) accounts for up to 10% of cases of neoplasm of the upper urinary tract. The “gold standard” management of upper tract TCC is nephroureterectomy. Technological innovations, miniaturisations and increased availability of energy sources such as Holmium laser fibers have improved the armamentarium of endoscopic management of upper tract TCC. Endoscopic management of upper tract TCC includes the percutaneous (antegrade) and retrograde approaches. Modern flexible ureterorenoscopy allows retrograde approach to small (<1.5cm), low grade and noninvasive tumors, which is inaccessible to standard rigid ureteroscopes without breaching the urothelial barrier. In patients with large tumors or in whom retrograde access is difficult, the percutaneous approach to the renal pelvis, although more invasive, provides an alternative access and control. Both retrograde and percutaneous approaches allow instillation of various chemotherapeutic agents. Careful selection of patients is the key point in the successful endoscopic management of upper tract TCC. Patient selection is based on tumor size, grade and multifocality and other patient factors such as comorbidities, single kidney, post kidney transplant and patient choice. Both motivation and compliance of patients are needed for long-term successes. However, until large randomized trials with long term follow-up are available, endoscopic management of upper tract TCC should be reserved for only selected group of patients. This review summarizes the current techniques, indications, contraindications and outcomes of endoscopic management of UTTCC and the key published data.

## INTRODUCTION

Upper urinary tract transitional cell carcinomas (TCC) are defined as tumors located anywhere on the urothelial lining between the renal calyces and vesicoureteric junction (VUJ) of the distal ureter. They are less common than TCCs that originate in the bladder, though the true incidence is difficult to identify due to epidemiological data often merging tumors of the renal pelvis with renal cell carcinoma statistics. Also, there is considerable geographic variability, with the highest reported incidence occurring in Balkan countries, associated with a degenerative interstitial nephropathy.[1] Other risk factors include smoking, male gender, multifocal bladder cancer and chronic analgesic abuse (phenacetin).[2] Staging and grading of upper tract TCC is similar to that of bladder cancer, though due to the relative thinness of the ureter’s muscle layer, ureteric tumors are more likely to be invasive at presentation.[3] Non-transitional cell types are uncommon, and may be associated with long-standing infected staghorn calculi (squamous cell carcinoma).

Upper tract TCC most commonly presents either with macroscopic or microscopic haematuria, or is discovered during follow-up imaging of patients with bladder TCC. Upper tract TCCs typically appear as a filling defect (that may cause obstruction) on intravenous pyelography, delayed phase CT urogram or retrograde urography. The bladder and contralateral tract must be carefully examined due to the significant incidence of multiple lesions. The role of pre-treatment histological diagnosis with cytology or ureteroscopy and biopsy is controversial due to reported poor sensitivity for low grade tumors in the former, and a small size of tissue obtained in the latter modality. It is thought that ureteroscopy with brush or forceps biopsy is not mandatory, but should be used in cases where the diagnosis is in doubt, or the management would be significantly altered by ureteroscopic findings.[4]

## NON-ENDOSCOPIC SURGICAL MANAGEMENT

Although the purpose of this review is to describe the current practice of endoscopic treatment of upper tract TCC, for completeness, the indications of non-endoscopic surgical treatment will be summarized. Historically, the “gold standard” treatment for upper tract TCC in suitable patients is nephro-ureterectomy, with resection of a cuff of bladder around the VUJ. Particular indications include muscle invasive or high grade TCC.[5] The surgical procedure has been described as open, laparoscopic with open excision of the bladder cuff, hand assisted laparoscopic, pure laparoscopic and robot-assisted laparoscopic. Although there are no prospective randomized controlled trials comparing the above, a recent review article investigated the retrospectively available data comparing open and laparoscopic nephro-ureterectomy in 1249 patients, and found that the short-term oncologic data was comparable, bearing in mind the possible selection bias of treatment modality.[6] A series of 252 patients found that outcome is highly dependent upon pathologic stage and grade, with five-year actuarial survival rates of just 50% for pT3 lesions, and under five per cent for pT4 tumors, compared to >90% for pTa and pT1tumors.[7] A kidney-sparing approach is possible for lesions of the distal ureter (e.g. distal ureterectomy with the options of psoas muscle hitch or Boari flap neocystotomy. The relative and absolute contra-indications to radical surgery, which may encourage a less invasive, endoscopic approach, are shown in Table 1.

**Table 1:** Relative and absolute contraindications to radical surgery

Relative contraindications	Absolute contraindications
Small, single distal ureteric tumor in a patient with mild to moderate comorbidity	Severe comorbidity precluding general anesthesia
Moderate comorbidity in a patient with a non-invasive, low grade tumor	Patient refusal of radical surgery
Poorly functioning contralateral kidney	Poorly functioning contralateral kidney in a patient who refuses or is not fit for dialysis

## ENDOSCOPIC MANAGEMENT

### Indications

We are in the modern era of the ageing patient; in the UK, the number of people aged over 65 exceeds 10 million, and worldwide, by 2025, the number of people worldwide aged over 60 will exceed one billion.[8,9] This transcribes in clinical practice to an increasing prevalence of elderly patients with significant comorbidities, and a greater importance of decision-making regarding risks versus potential benefits of invasive procedures that require general anesthesia. The advent of improved technology, especially in the fields of fiber-optics, the miniaturization of instruments and availability of increasingly flexible and maneuverable energy sources such as laser fibers have improved the armamentarium of endoscopic treatments of upper tract TCCs. Endoscopic management of upper tract TCC is particularly suited to small (<1.5cm), low grade and noninvasive tumors, in comorbid patients who are at high risk for major radical surgery or who have compromised renal function (including a single functioning kidney), and patients who refuse radical surgery.

### Retrograde vs. antegrade vs. combined approaches

The feasibility of using an endoscopic approach to upper tract TCC is dependent on the location of the tumor(s), available expertise (e.g. expertise to create percutaneous access) and the availability of often expensive technical equipment (such as flexible ureterorenoscopes and laser generators). A retrograde ureteroscopic approach has the advantage of maintaining a closed system - i.e. the urothelial barrier is not breached, hence reducing the risk of tumor seeding. Also, this approach does not require the expertise of percutaneous access and formation of a tract, which have the associated risk of bleeding (including need for transfusion and rarely loss of renal unit), infection and damage to adjacent viscera including pneumothorax and bowel injury.[10]

A retrograde approach with a semi-rigid ureteroscope is usually the most appropriate endoscopic modality to access ureteric tumors. If accessibility to the upper ureter is difficult or the tumor is located in the renal pelvis, flexible ureterorenoscopy is deployed. Modern flexible ureterorenoscopes allow deflection angle of up to 270°. However, passing a laser fibre leads to loss of deflection of between 4 and 10%, and causes a reduction in the irrigation volume of 54%.[11]

An antegrade approach via percutaneous access to the collecting system was first described by Tomera *et al*, with a subsequent larger series reported by Smith *et al*.[12,13] Percutaneous surgery is advocated to treat larger tumors located in the renal pelvis or proximal ureter and in patients with failed ureteroscopic access (including difficult tumors in a lower pole calyx).[12] A further indication of antegrade treatment is in patients with previous urinary diversion, most commonly prior cystectomy for bladder TCC, though retrograde access may be possible but often technically challenging.[14,15] A combined retrograde and antegrade approach may be necessary in patients with multifocal upper tract TCC or a renal pelvic tumor with superficial extension down the ureter.[16]

## URETEROSCOPIC TECHNIQUES

A retrograde approach using a semi-rigid ureteroscope to treat localized low-grade upper tract tumors was first described by Huffman *et al*.[17] Informed consent for the procedure is obtained after explaining the risks of ureteric perforation, avulsion and stricture, hemorrhage, infection and residual tumor. Other possible disadvantages of ureteroscopic management include - the need for multiple treatment, understaging, difficulty in obtaining enough tissue for accurate diagnosis and grading, and an occasional inability to gain access. Under general or spinal anesthesia, prophylactic antibiotics are administered according to local policy, e.g. intravenous Gentamycin 120mg. A cystoscopy and retrograde pyelogram are performed, and urine or a ureteric washout may be sent for cytological analysis. In the cases of renal pelvic TCC, an image of the contrast filled collecting system to be used as a ‘road map’ of the calyces is saved. A guide wire may be passed to the renal pelvis with fluoroscopic imaging guidance, though some centers prefer not to use a guide wire as it may dislodge tumor, and the resulting hemorrhage may reduce visibility. In the case of flexible ureterorenoscopy, an access sheath can aid the repeated passage of the instrument, and can be helpful to decrease irrigation pressures during long procedures.[18] There have been several methods described to endoscopically manage the tumor. First, tumors may be debulked with biopsy forceps, taking care not to resect deep tissue or to avulse the ureter. Alternative physical methods of debulking are extraction using a stone basket (with care), or, particularly in the pre-laser era, resection with an ureteroscopic resectoscope.[16,19] Sampled tissue is sent for histopathological analysis. Second, an energy source is applied to fulgurate the base of the tumor. Here, options are electrocautery via a 2-3Fr ‘Bugbee’ electrode, or vaporization with laser energy.

The two commonly described laser sources are holmium:yttrium-aluminium-garnet (Ho:YAG) or neodymium:yttrium-aluminium-garnet (Nd-YAG). Characteristics of the energy sources are depicted in [Table 2]. A ureteric stent is placed at the end of the procedure, and left until a ‘second-look’ repeat procedure is performed, usually at 6-12 weeks.

**Table 2:** Characteristics of energy sources used to treat upper tract transitional cell carcinoma

	Electrofulgaration	Ho:YAG laser	Nd-YAG laser
Diameter of electrode/fi bre	2-3Fr	200µm or 360µm	200µm or 360µm
Penetration	Variable	0.5mm (ablation/vaporization)	4-6mm (coagulative necrosis)
Advantages	Cheap, readily available	Precise, good hemostasis, less risk of stricture	Good hemostasis, less risk of stricture
Disadvantages	Risk of stricture, especially with circumferential use. Less flexible than laser fibers.	Expensive, coagulation of tumor may mask viable tumor at base	Expensive, vision may be obscured by debris
References	16, 20	16, 21, 22	16, 21-23

## PERCUTANEOUS TECHNIQUES

Prior to the procedure, informed consent for the procedure is obtained, with an explanation of the risks of hemorrhage (transfusion rate of up to 37%), damage to the kidney or adjacent viscera, pneumothorax, infection, residual tumor, pelvic-ureteric junction (PUJ) obstruction/stricture and (rarely) tumor seeding.[19,24,25] Urine should be cultured prior to the procedure, and prophylactic antibiotics are administered according to local policy. Under general anesthesia, a cystoscopy and retrograde pyelography are performed, and urine or a ureteric washout may be sent for cytological analysis. The patient is rotated into a prone or semi-prone position. Percutaneous access is performed either by the operating urologist or a radiologist, depending on local expertise. The choice of calyceal puncture is important as it significantly affects the technical ease of the procedure. Puncture site is influenced by similar principles as in percutaneous stone surgery, for example a solitary tumor in a calyx can be directly accessed.[19] Following successful puncture of the required area of the collecting system, a guide wire is passed, and the tract is dilated to accommodate a 30Fr Amplatz sheath either with balloon dilatation or serial dilators. The guide wire is then exchanged for a stiff guide wire, and the ureteric catheter may be grasped and pulled out of the sheath. The renal pelvis, proximal ureter and calyces are then thoroughly examined with a nephroscope or flexible endoscope. Tumor tissue is removed with coldcup biopsy forceps or a loop resectoscope and is sent for histopathological analysis. The remaining tumor is then ablated and hemostasis achieved either with electrocautery (e.g. using the rollerball element) or laser energy as described above and in Table 2. At the end of the procedure, a nephrostomy tube is left *in situ*, beyond the PUJ. Smith and co-workers advocate performing a nephrostogram within 24 hours to exclude extravasation.[19] Although some authors have reported the use of immediate postoperative irrigation of the collecting system and percutaneous tract with the antimetabolite 5-fluorouracil (5-FU) with the aim of reducing tumor seeding, others recommend deferring adjuvant therapy for period of one to two weeks to allow the urothelium to heal.[19,23] A ‘second look’ repeat procedure is recommended within one to two weeks to identify, biopsy and remove or destroy any suspicious areas that may represent residual tumor (particularly the base of the lesion), or missed lesions. A smaller (8Fr) nephrostomy tube may then be placed for subsequent intra-pelvic instillations as described below.

## ADJUVANT THERAPY

The aim of instilling chemo- or immunotherapeutic agents post resection is to reduce tumor recurrence. Agents can be administered via a percutaneous nephrostomy after percutaneous management, or via an ureteric catheter following ureteroscopic treatment. Essentially, the same agents have been used to treat the upper tract as have been used in the bladder, namely mitomycin C, thiotepa and Bacillus Calmette-Guérin (BCG). The fact that many different agents and different timing protocols have been reported demonstrates that there is no standardized regime established from prospective randomized trials.[26] Suggested regimes and complications are illustrated in Table 3. A recently published large series of treating 133 renal units over a 20-year period demonstrated no benefit in reducing recurrence or progression with adjuvant BCG following percutaneous resection of upper tract TCC.[30] If adjuvant BCG is given, it is recommended that to avoid systemic absorption and possible sepsis, instillation of agents to the upper tract should be performed under low pressure (25 cm water) and in the absence of infection.[16] Close observation after BCG is recommended for 24 hours; with immediate cessation of therapy and prompt initiation of anti-tuberculous treatment if systemic symptoms develop.[31]

**Table 3:** Regimes and complications of adjuvant therapy

	Mitomycin C	BCG	Thiotepa
Timing, number of patients	Immediately after first procedure, 5 patients[27]	Immediately after first procedure, 9 patients[27]	Immediately after first procedure, 4 patients[27]
	40mg over 30 min after first procedure, 19 patients[28]	Weekly for 6 weeks, 37 patients[29]	
		Weekly for 6 weeks, two weeks after percutaneous resection[30]	
Complications	Nil[27,28]	Nil[27], BCG inflammation (1 patient) severe septicaemia (2 patients)[29]	Nil[27]

## RESULTS

Although there have been no published prospective, randomized trials regarding the endoscopic management of upper tract transitional cell carcinoma, there is a wealth of retrospective experience published as case series and review articles.[32,33] Comparison of the efficacy of ureteroscopic versus percutaneous data is difficult due to differing patient populations (including tumor size, stage and grade) and differences in measuring outcome. Table 4 and 5 display results from the major contemporary series of ureteroscopic and percutaneous management, respectively. Close surveillance of both the bladder and upper tract(s) is mandatory due to the high rate of recurrence; indeed authors suggest that nearly all patients will ultimately recur. An example of a follow-up regime is cytology with rigid cystoscopy and (bilateral) ureteroscopy every three months for the first year, then life-long surveillance.[42]

**Table 4:** Published data of the ureteroscopic management of upper tract TCC

Author, year reference	Number of renal units	Mean tumor size (cm)	Recurrence rate (%)	Mean follow up (months)	Comments
Rouprêt 2006[34]	27	1.4	44	52	No adjuvant treatment, Ureteric perforation in 2 patients
Thompson 2008[35]	83	0.9	55	55	Data included 7 patients treated with percutaneous approach. 33% eventually had nephro-ureterectomy
Daneshmand 2003[36]	26	No data (ND)	88	28	
Johnson 2005[21]	35	2.2	68	32	All patients had low grade tumors
Suh 2003[37]	18	1.3	75	15	Two procedures abandoned due to ureteric perforation. 3 eventually had nephro-ureterectomy
Sowter 2007[38]	37	All <2cm	74	42	One ureteric perforation, 4 strictures

**Table 5:** Published data of percutaneous management of upper tract transitional cell carcinoma

Author, year reference	Number of renal units	Mean tumor size (cm)	Recurrence rate (%)	Mean follow up (months)	Comments
Rouprêt 2007[39]	24	1.8	33	62	One iatrogenic colonic injury, 3 required transfusion
Suh 2003[37]	19	2.9	100	16	One required blood transfusion, 1 pneumothorax. 4 eventually had nephroureterectomy
Clark 1999[14]	17	No data (ND)	33	20.5	16 patients had 6 weeks of adjuvant BCG
Liatsikos 2001[19]	69	ND	36	49	37% transfusion rate. 30 patients had 6 weeks of adjuvant BCG.
Goel 2003[40]	22	ND	55	64	Mitomycin C or epirubicin given post operatively. Two patients died of renal failure.
Palou 2004[41]	34	ND	41	51	Adjuvant chemo- or immunotherapy used. 26% required nephro-ureterectomy.

## CONCLUSIONS

Technological advances have led to an increase in endoscopic management of upper tract TCC. However, all published data are small, retrospective with a short follow-up and high recurrence rate. Careful selection of patients is the key message. Endoscopic management of upper tract TCC is particularly suited to small (<1.5cm), low grade and noninvasive tumors, in comorbid patients who are at high risk for major radical surgery or who have compromised renal function (including a single functioning kidney), and patients who refuse radical surgery. For high grade tumors, endoscopic management is essentially palliative. Patients should also be highly motivated and compliant as lifelong surveillance is necessary. Until large randomized trials with long follow-up are available, endoscopic management of upper tract TCC cannot be recommended as an alternative to nephroureterectomy.
